# Integrated Extraction and Structural Engineering of Chitin from Crayfish Shell Waste Using Alkaline Deep Eutectic Solvents Toward Facile Enzymatic Deacetylation

**DOI:** 10.3390/foods15071159

**Published:** 2026-03-30

**Authors:** Shengyu Yang, Qingqing Xiao, Kaige Chen, Haojie Zhang, Jun Cai, Zexin Zhao

**Affiliations:** Key Laboratory of Fermentation Engineering (Ministry of Education), Hubei Key Laboratory of Industrial Microbiology, National “111” Center for Cellular Regulation and Molecular Pharmaceutics, Cooperative Innovation Centre of Industrial Fermentation (Ministry of Education & Hubei Province), Hubei University of Technology, Wuhan 430068, China

**Keywords:** deep eutectic solvent, crayfish shell waste, extraction, structural engineering, chitin deacetylase, molecular dynamics simulation

## Abstract

Development of green and efficient technologies for valorizing crayfish shell waste is crucial for enhancing industrial value. This study presents an integrated strategy for the extraction and structural engineering of chitin using a novel alkaline deep eutectic solvent (DES) system composed of lysine and monoethanolamine (LysMEA), which enables the simultaneous deproteinization and architectural modification of chitin. Following mild demineralization, the optimized process yielded chitin with 97.1% purity and a high molecular weight of 209.3 kDa. DES demonstrated considerable reusability and decolorization capability. Structural characterization revealed that the LysMEA system effectively engineered the chitin architecture, resulting in lower crystallinity and a larger surface area compared to conventional methods. This engineered structure rendered the chitin highly accessible to enzymes. Consequently, the chitin extracted by LysMEA exhibited superior reactivity, achieving a deacetylation degree of 63.7% when catalyzed by *Bacillus aryabhattai* chitin deacetylase, significantly outperforming chitin obtained via acid-alkali or acidic DES methods. Molecular dynamics simulations elucidated the mechanism, showing that lysine and monoethanolamine molecules penetrated the chitin fiber bundles at high temperatures, weakening interchain hydrogen bonds and partially separating the chains. This work provides a green route for producing enzymatically reactive chitin, demonstrating the potential of solvent-based structural engineering in biocatalytic valorization.

## 1. Introduction

Chitin, the second most abundant natural polymer on Earth after cellulose, is a unique nitrogen-containing polysaccharide composed of N-acetyl-D-glucosamine units linked by β-1,4 glycosidic bonds [1]. Widely distributed in exoskeletons of crustaceans, insect cuticles, and the cell walls of fungi and algae, chitin has garnered significant attention owing to its advantageous properties, including renewability, biodegradability, biocompatibility, and pleiotropic bioactivities [2,3,4]. Notably, its deacetylated derivative, chitosan, exhibits superior solubility, thereby unlocking broader applications in food, biomedical, agricultural, and cosmetics sectors [5,6]. With the rapid expansion of the global aquaculture processing industry, the disposal of processing waste has become a pressing environmental concern. For instance, in China, the annual processing volume of red swamp crayfish (*Procambarus clarkii*) reached 1.5 million tons by 2024, generating approximately 70% waste by weight, much of which contains valuable chitin. Therefore, efficient extraction and conversion of chitin from crustacean waste not only have the potential to generate significant economic value but also support the establishment of a circular economy.

In crustacean exoskeletons, chitin is embedded within a mineral–protein matrix [7], requiring demineralization (DM) and deproteinization (DP) for extraction, with decolorization often necessary to achieve high purity. Although the conventional acid-alkali process remains the dominant industrial method, it suffers from severe drawbacks, including a heavy environmental burden and reduced product quality [8]. Environmentally friendly biological approaches, such as microbial fermentation and enzyme-assisted extraction, have been explored for chitin extraction from crustacean wastes [9,10,11]. However, their large-scale application is hindered by prolonged processing times, high operational costs, and complexity. Emerging green solvent systems, particularly ionic liquids (ILs) and deep eutectic solvents (DESs), have attracted growing interest as more sustainable and efficient alternatives for chitin extraction [12,13,14,15]. Unfortunately, regardless of the extraction route, the resultant chitin invariably exhibits a dense, highly crystalline structure. This inherent recalcitrance severely limits the accessibility of chemical reagents and enzymes, posing a major bottleneck for downstream processing.

This structural barrier is particularly evident in chitosan production. While harsh thermal-alkaline treatment remains the industrial standard for chitin deacetylation, it suffers from significant sustainability issues, including high energy consumption, excessive alkali use, and alkaline waste generation, and yields structurally heterogeneous chitosan due to non-selective reactions [16]. In contrast, enzymatic deacetylation using chitin deacetylases (CDAs) offers a promising green alternative, enabling site-specific and controlled deacetylation under mild conditions. This approach preserves the glycosidic backbone and allows precise modulation of the degree of deacetylation, facilitating the production of tailor-made chitosan with optimized functional properties [17]. However, the practical implementation of enzymatic processes is severely constrained by the low accessibility of CDAs to the tightly packed chitin structure, wherein dense crystalline regions impede enzyme diffusion and binding, leading to slow reaction kinetics and incomplete conversion [18].

To overcome this limitation, various physical pretreatment methods, including sonication [19], microwave treatment [19], steam explosion [20], superfine grinding [21] and supercritical CO_2_ technology [22], can effectively reduce crystallinity and increase specific surface area. While effective in enhancing substrate accessibility, these techniques often result in heterogeneous molecular weight distributions and entail high energy input, making them impractical for large-scale application. Alternative approaches, such as dissolving chitin in alkali/urea systems followed by rapid precipitation, can reduce crystallinity but are too labor-intensive and difficult to scale up for industrial production [23]. Although certain ILs have been shown to effectively disrupt the crystalline structure of chitin during extraction [24,25], the potential of DESs in this regard remains less explored. Notably, some acidic DESs may even increase chitin crystallinity by degrading amorphous regions [26,27,28]. In contrast, alkaline DESs have demonstrated success in pretreating lignocellulosic biomass by disrupting hydrogen bonding networks and removing lignin, thereby enhancing cellulose accessibility [29,30,31]. Encouragingly, recent studies have extended their application to chitin-rich materials. Lv et al. successfully extracted β-chitin from squid pens using an alkaline DES formed by K_2_CO_3_ and glycerol [32], while Zhang et al. developed ternary alkaline DESs, containing amino acids, urea, and 1,8-diazabicyclo [5.4.0] undec-7-ene (DBU), capable of producing low-crystallinity chitin from crayfish shell [33]. These studies highlight the potential of alkaline DES systems for the integrated extraction and structural engineering of chitin from chitin-rich bioresources.

While alkaline DESs, including amino acid/ monoethanolamine (MEA) systems (e.g., aspartic acid, asparagine, glutamine, proline, and lysine with MEA) and choline chloride (ChCl)-based systems (e.g., ChCl/MEA and ChCl/triethanolamine (TEA)) [30,34,35], have demonstrated significant potential in the pretreatment of various biomass feedstocks, their capacity for chitin extraction from crustacean shell waste remains largely unexplored. To bridge this gap, this study investigates the efficacy of these solvents for the integrated valorization of crayfish shell waste, specifically assessing the performance of both the previously reported K_2_CO_3_/glycerol DES and the newly designed lysine/TEA system. Following the comparative evaluation, the composition ratios and extraction conditions were systematically optimized for the most effective system to maximize chitin yield and purity. The resulting chitin was comprehensively compared with those obtained via acidic DES (ChCl/malic acid) and traditional acid-alkali treatments. Furthermore, their accessibility to well-characterized chitin deacetylases from *Aspergillus nidulans*, *Bacillus aryabhattai* and *Saccharomyces cerevisiae* (AnCDA, BaCDA and ScCDA, respectively) [36,37,38] was examined. The reusability of the alkaline DES was also evaluated. Moreover, the underlying mechanism by which alkaline DES enhances chitin accessibility was elucidated through molecular dynamics simulations. This work thus aims to establish a holistic strategy for the sustainable production of enzymatically reactive chitin from crustacean shell wastes.

## 2. Materials and Methods

### 2.1. Materials, Plasmids and Strains

Red swamp crayfish shells were provided by Chengfeng Biotechnology Co., Ltd. (Jingmen, Hubei, China). Glycerol, ChCl, MEA, TEA, L-aspartic acid (Asp), L-asparaginate (Asn), L-glutamine (Gln), L-proline (Pro) and L-lysine (Lys), lithium chloride, and N,N-dimethylacetamide (DMAc) were purchased from Aladdin (Shanghai, China). Racemic malic acid (MA) was supplied by TCI (Shanghai, China). Antibiotic kanamycin sulfate (Kan) and isopropyl β-D-1-thiogalactopyranoside (IPTG) were purchased from Sangon (Shanghai, China). Commercial chitin, paranitroacetanilide and paranitroaniline were purchased from McLean (Shanghai, China). Commercial chitosan and other reagents were of analytical grade and provided by the China National Pharmaceutical Group Chemical Reagent Co., Ltd. (Shanghai, China). The pET28a plasmids, containing genes that encode AnCDA, BaCDA and ScCDA, respectively, were synthesized by Sangon. *Escherichia coli* (*E. coli*) BL21 (Weidi Biotechnology Co., Ltd., Shanghai, China) was used as the expression host.

### 2.2. Preparation of Crayfish Shell Powder and Acid-Mediated Demineralization (DM)

The red swamp crayfish shells were thoroughly washed with deionized water, dried in an oven at 60 °C, and then ultra-finely ground with a mill (RT-25, Xinzhen Technology Co., Ltd., Taiwan, China) to gain 60-mesh crayfish shell powder (CSP, containing 24.8% chitin, 26.5% protein and 48.7% mineral content). In order to demineralize it, the prepared CSP was treated with 5% (*w*/*v*) HCl at 40 °C for 1 h. The precipitate was collected by centrifugation at 8000× *g* for 8 min and subsequentially washed with deionized water until neutral pH was achieved. Finally, the demineralized crayfish shell powder (DM-CSP) was obtained by drying at 60 °C.

### 2.3. Synthesis of DESs

To prepare each DES system, a hydrogen bond acceptor (HBA) and donor (HBD) were added to a beaker in a certain molar ratio, then mixed at 100°C with continuous stirring until a transparent liquid was formed. This process is driven by a eutectic effect, wherein proton transfer and the formation of an extensive hydrogen-bonding network between the HBA and HBD disrupt the crystalline lattices of the individual components, thereby significantly lowering the melting point of the mixture [39]. The unoptimized molar ratio of components for each DES system used in the study is shown in Table 1.

### 2.4. Viscosity Determination of DESs

The viscosity of DESs was evaluated using a Haake Mars 60 rheometer (Thermo Scientific, Karlsruhe, Germany) with a concentric cylinder double-gap geometry. Approximately 1 mL of the sample was loaded in the gap (0.1 mm) and measured with shear mode (0.01–100 s^−1^) at 25 °C.

### 2.5. Chitin Extraction from DM-CSP/CSP

#### 2.5.1. Solvent Screening

Due to the limited DM capability of alkaline DESs observed during initial screening, a two-step strategy was adopted. First, CSP was subjected to acid-mediated demineralization to obtain DM-CSP (as described in Section 2.2). For screening the alkaline DESs (Table 1), DM-CSP was treated with each solvent system at a solid-to-liquid ratio of 1:15 at 80 °C for 6 h. After cooling, deionized water was added to reduce viscosity, followed by centrifugation (8000× *g*, 8 min) and washing to neutrality. For comparison, a one-step chitin extraction using CSP as a substrate was also attempted. Instead of DM-CSP, CSP was added to alkaline DESs, and, with the exception of incubation at 100 °C for 12 h, the subsequent steps followed the procedure described above.

#### 2.5.2. Process Optimization and Comparative Preparation

Based on the screening results, the LysMEA system was selected for further optimization of the molar ratio, temperature, time, and solid-to-liquid ratio. Under the final optimized conditions (120 °C, 12 h, HBA/HBD molar ratio 1:8 and solid-to-liquid ratio 1:20), chitin was extracted and designated as LM-chitin.

For comparison, chitin extracted from CSP by a traditional acid-alkali method followed the process described by Saravana et al. [40]. The prepared DM-CSP was mixed with 9.1% (*w*/*w*) NaOH solution at a solid-to-liquid of 1:20 and heated at 90 °C for 3 h. The resulting solid was collected by centrifugation at 8000× *g* for 8 min and then washed with deionized water until the wash solution reached neutrality. Purified chitin was obtained by drying at 60 °C and designated as AA-chitin.

Additionally, the method described by Feng et al. [41] was employed to extract chitin from CSP using the ChClMA DES system. Briefly, CSP was added into the DES system at a solid-to-liquid ratio of 1:20, and incubation of the mixture was performed at 130 °C for 3 h. The product was collected by centrifugation (8000× *g* for 8 min) and washed with deionized water until neutral pH was reached. For further deproteinizing, the solid, dried to constant weight, was treated with a NaOH solution under the conditions described above. Centrifugation, washing and drying were then sequentially executed to gain purified chitin (designated as CM-chitin).

### 2.6. Evaluation of Chitin Purity and Yield

Determining the mineral content of each sample was done according to the method mentioned by Rasweefali et al. [42]. The sample was heated to complete carbonization in a crucible and repeatedly incinerated in a resistance furnace at 600 °C for 4 h until a constant weight was achieved. The mineral weight of the sample was obtained by weighing, and the DM rate is calculated as follows (Equation (1)):

(1) $$\mathrm{DM}\;\mathrm{rate}\;(\%)\;=\;\frac{M_{0}-M_{1}}{M_{0}}\;\times 100\%$$

M_0_ and M_1_ represent the mineral weights (mg) of the initial CSP and the treated sample, respectively.

To measure the protein content, the sample was dissolved in 5% (*w*/*v*) NaOH at a solid-to-liquid ratio of 1:20 and heated at 95 °C for 2.5 h. The supernatant was separated by centrifugation (8000× *g* for 8 min) and then the protein content of the sample was measured by the Bradford method [43]. The DP rate is calculated as follows (Equation (2)):

(2) $$\mathrm{DP}\;\mathrm{rate}\;(\%)\;=\;\frac{P_{0}-P_{1}}{P_{0}}\;\times 100\%$$

P_0_ and P_1_ represent the protein weights (mg) of the initial CSP and the treated sample, respectively.

The chitin purity (P_c_) of each sample is calculated as follows (Equation (3)) [40]:

(3) $$P_{c}\;(\%)\;=\;\frac{M_{T1}-M_{1}-P_{1}}{M_{T1}}\;\times 100\%$$

M_T1_ represents the total weight (mg) of the processed sample.

The chitin yield (Y_c_) is calculated as follows (Equation (4)) [40]:

(4) $$Y_{c}\;(\%)\;=\;\frac{M_{T1}\times P_{c1}}{M_{T0}\times 24.8\%}\;\times 100\%$$

P_c1_ represents the chitin purity of the processed sample, M_T0_ represents the initial weight (mg) of CSP, and 24.8% is the chitin content of CSP.

### 2.7. Recycling of LysMEA DES

Extraction of chitin by LysMEA DES was performed under optimized conditions. After product separation, the appropriate amount of ethanol was added to the supernatant to precipitate the protein from DM-CSP. The liquid portion, collected by centrifugation (8000× *g* for 10 min), was subjected to reduced pressure evaporation at 45 °C to remove ethanol and water. The regenerated DES was then reused in the next extraction cycle.

### 2.8. Characterization of Chitin Sample

#### 2.8.1. Molecular Weight

The intrinsic viscosity ([η]) of each chitin sample (dissolved in 5 wt% LiCl/DMAc) was measured using a Ubbelohde viscometer at 30°C. The average molecular weight (M_w_) of samples prepared by various approaches was calculated by the Mark–Houwink–Sakurada equation [44] (Equation (5)):

[η] = KM_W_^α^ (5)

where K = 7.6 × 10^−5^ dL/g and α = 0.95.

#### 2.8.2. ^13^C NMR Spectroscopy

Each dry chitin sample was analyzed using a JNM-ECZL600G spectrometer (Jeol, Tokyo, Japan) operating at 150.76 MHz with 1800 scans for ^13^C CP/MAS solid-state NMR spectra at 25 °C. Cylindrical 3.2 mm zirconia rotors were employed and spun at 15 kHz. Determination of the DD using ^13^C NMR spectroscopy is performed with the following equation [45] (Equation (8)):

(6) $$\mathrm{DD}\;(\%)\;=(1-\frac{\int C-{CH}_{3}}{\frac{1}{6}(\int C1+\int C2+\int C3+\int \;C4+\int C5+\int C6)})\times 100$$

#### 2.8.3. Fourier-Transform Infrared Spectroscopy (FTIR)

FTIR analysis of samples was carried out using a Nicolet iS10 spectrometer (ThermoFisher, Waltham, MA, USA) over a wavenumber range of 500–4000 cm^−1^ with a resolution of 4 cm^−1^. The deacetylation degree (DD) of chitin samples is calculated by the empirical formula [46] (Equation (6)):

(7) $$\mathrm{DD}\;(\%)\;=\;1-\frac{\frac{A_{1320}}{A_{1420}}-0.3822}{0.03133}$$

#### 2.8.4. X-Ray Diffraction (XRD)

XRD analysis was performed using a Miniflex 600 (Rigaku, Tokyo, Japan) instrument with CuKα radiation (λ = 1.5406) (40 kV, 30 mA). The 2θ angle was scanned from 5° to 45° with a scanning speed of 5°/min. The crystalline index (CrI) is calculated as follows (Equation (7)):

(8) $$\mathrm{CrI}\;(\%)\;=\;\frac{I_{110}-I_{am}}{I_{110}}\times 100\%$$

I_110_ and I_am_ represent the maximum intensity at 2θ ≈ 20° and 2θ ≈ 16°, respectively.

#### 2.8.5. Scanning Electron Microscope (SEM)

To observe the morphology, each sample was covered with a thin gold layer using a Bio-Rad SC-502 sputter coater (Hertfordshire, UK) and examined via a JSM-6390LV SEM (Jeol, Tokyo, Japan).

#### 2.8.6. Brunauer–Emmett–Teller Analysis (BET)

Gas adsorption isotherms were measured using an ASAP 2460 surface area and porosity analyzer (Micrometrics, Londonderry, NH, USA). Prior to measurement, the sample was degassed at 100 °C for 12 h.

### 2.9. Production of CDAs

The recombinant pET28a plasmids harboring genes encoding AnCDA (Uniprot ID: Q5AQQ0), BaCDA (Uniprot ID: A0A7W3NGR0) and ScCDA (Uniprot ID: Q06703) were transformed into *E. coli* BL21 to construct the expression strains. After coating separation, single colonies of the recombinant cells were randomly picked from Luria–Bertani (LB) medium plates containing 50 μg/mL Kan to LB broth and grown at 37 °C and 220 rpm in a shark bath. Until the OD_600_ of the broth achieved about 0.6 mM, IPTG was added at a final concentration of 0.2 mM to start the expression of CDA at 20 °C for 20 h. Then, the cells were collected from the culture broth by centrifugation at 4 °C and 10,000× *g* for 15 min, and resuspended with a 50 mM phosphate buffer (pH 7.4). Target proteins were released from the cells by ultrasonication, and the supernatant after centrifugation (10,000× *g*, 15 min at 4 °C) was filtered through a 0.22 μm membrane and collected as CDA solutions. CDA activity was determined by colorimetric techniques using paranitroacetanilide as the substrate [45]. One unit of enzyme activity is defined as the amount of enzyme required to release 1 mmol of paranitroaniline per minute at 37 °C.

### 2.10. Enzymatic Deacetylation

To test the deacetylation effect of CDA on various substrates, 50 mg of the chitin sample was mixed with 5 mL of 50 mM phosphate buffer (pH 7.4) containing 20 U of CDA and incubated at 30 °C with shaking at 400 rpm for 12 h. The reaction was terminated by inactivation with boiling water. Then, the precipitate was centrifuged and washed and finally freeze-dried to obtain the deacetylated product.

### 2.11. Measurement of Solubility of the Deacetylated Product

Determination of product solubility was performed according to the method described by El-araby et al. [47]. Briefly, 0.5 g of chitosan was mixed with 10 mL of 1% acetic acid solution, and the mixture was stirred continuously at 60 °C for 24 h. The precipitate obtained by centrifugation (5000× *g* for 8 min) was dried to a constant weight, and the solubility of product (S_p_) is calculated as follows (Equation (8)):

(9) $$S_{p}\;=\;\frac{m_{0}-m_{1}}{m_{0}}$$

m_0_ and m_1_ represent the initial and residual weight of the sample, respectively.

### 2.12. Computational Methods

The optimized structures of Lys, MEA, L-malic acid, D-malic acid and choline were acquired from the ATB website (https://atb.uq.edu.au/index.py). All models were constructed by Packmol [48], including the start model for the chitin fiber bundle (consisting of twelve chitin decamers, see Figure S1A) and the models immersing the compact fiber bundle (Figure S1B) into water, LysMEA and ChClMA. Molecular dynamics simulations (MD) were conducted using a GPU-accelerated engine provided by GROMACS 2019 [49]. A 200 ns MD simulation was performed for each system to investigate the ability of different solvents at different temperatures to disaggregate chitin fiber in silico. The detailed processes and parameters are provided in the Supplementary Materials.

### 2.13. Statistical Analysis

All experiments were performed at least three times. All results are reported as the mean values ± standard deviations (SD).

## 3. Results and Discussion

### 3.1. Screening of Efficient Alkaline DES for Chitin Extraction

**Figure 1:**
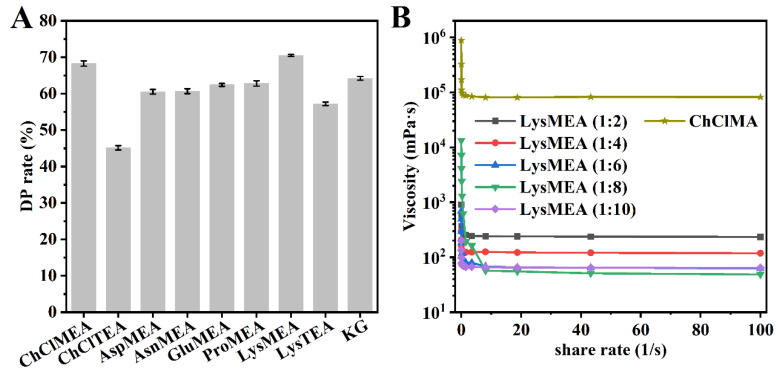
Deproteinization efficiency of alkaline DESs (**A**) and the viscosity of LysMES at different composition ratios and ChClMA (**B**).

Nine alkaline DESs (designation and composition of each system shown in Table 1) were initially screened for their ability to extract chitin from CSP. Regrettably, even under harsh conditions (100 °C, 12 h), the maximum DM rate achieved was only 65.4%, although their DP rate can reach at least 88.5% (Figure S2). This result highlights a current limitation of the alkaline DES system. While highly effective for deproteinization (DP), it lacks the proton-donating capacity required for efficient calcium carbonate dissolution. In a recent study, a two-step method consisting of alkaline DES extraction followed by a lactic acid treatment was applied to prepare chitin and calcium lactate, also indicating the limited DM ability of alkaline DESs [33]. Consequently, to achieve suitable high-purity chitin, we adopted a two-step approach. A mild HCl pre-treatment was employed specifically for DM, followed by the solvent treatment for DP. The demineralized CSP (DM-CSP, DM rate: 98.3%) was mixed with alkaline DESs at 80 °C for 6 h to further purify chitin. Among the alkaline DESs, LysMEA and ChClMEA displayed higher DP efficiency, achieving a DP rate of 70.5% and 68.3%, respectively (Figure 1A). The LysMEA system possessed the strongest alkalinity compared to other amino acid/MEA systems, which contributed to protein dissolution. The DP efficiency of the GK system was lower than that of LysMEA (Figure 1A), which can be attributed to the significantly higher viscosity of the GK system (>10^5^ mPa·S) [32]. This higher viscosity impeded the penetration of the solvents into the raw material and their interaction with protein. Substituting MEA with TEA resulted in a reduced DP ability for both Lys-based and ChCL-based DES (Figure 1A), due to the significantly higher viscosity of TEA compared to MEA [50]. Considering its superior DP efficiency compared to other alkaline DESs, the LysMEA system was selected for further optimization.

### 3.2. Optimization of the Extraction Conditions and Reuse of LysMEA DES

**Figure 2:**
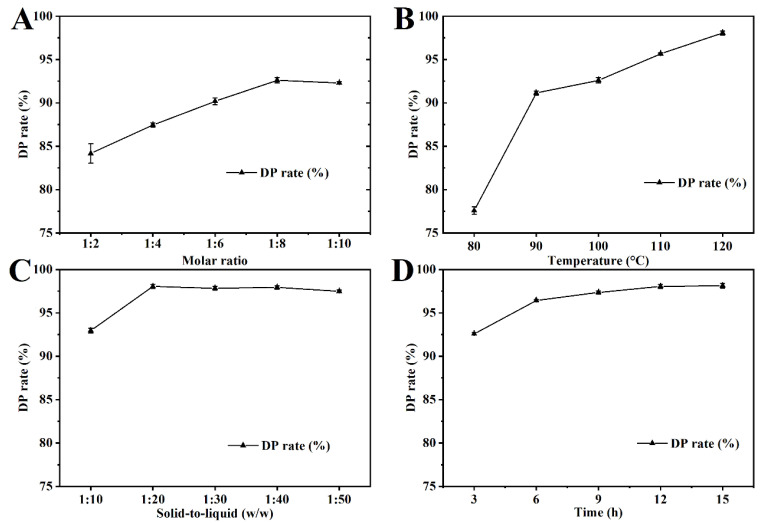
Single-factor optimization of chitin extraction conditions using LysMEA DES system. Effects of molar ratio of Lys/MEA (**A**), treatment temperature (**B**), solid-to-liquid ratio of raw material/DES (**C**), and treatment time (**D**) on deproteinization (DP) rate.

First, the effect of the Lys/MEA molar ratio on DP efficiency was investigated at a solid-to-liquid ratio of 1:20, under conditions of 100 °C for 12 h. As shown in Figure 2A, the DP rate increased as the ratio decreased from 1:2 to 1:8. A maximum DP rate of 92.6% was achieved after incubation in LysMEA with a molar ratio of 1:8. Further increases in the proportion of MEA led to a reduction in the DP efficiency of the system. Interestingly, with an increase in the proportion of MAE, the viscosity of the system initially decreased and then increased, reaching a minimum at a 1:8 ratio (Figure 1B). This phenomenon implies that when the molar ratio of Lys/MEA is below 1:8, a stronger interaction may form between the solvent molecules, resulting in an increase in the viscosity of the system. Therefore, other parameters were optimized at a Lys to MEA molar ratio of 1:8, and LysMEA will be referred to as this solvent system in subsequent sections.

The effect of temperature on the DP efficiency of LysMEA was also explored. The DP rate increased with the rise in processing temperature, achieving 98.1% when the temperature reached 120 °C (Figure 2B). Higher temperatures effectively reduce the viscosity of the DES system, thereby increasing the contact opportunities between the solvent and protein in DM-CSP, which facilitate protein dissolution. This trend is consistent with the results of a recent study that employed a ChCL-lactic acid DES system to extract chitin from *Hermetia illucens* pupae shells [51]. Although further increasing the temperature may slightly enhance efficiency, considering energy consumption, this study adopted a temperature of 120 °C for extraction.

Solid-to-liquid ratio of DM-CSP to LysMEA (*w*/*w*) and treatment time are other critical factors affecting DP efficiency of the DES system. As the solid-to-liquid ratio increased from 1:10 to 1:20, the DP rate of products sharply increased, but further increases did not result in a significant improvement (Figure 2C). The results indicate that an appropriate increase in the amount of LysMEA can enhance the contact area between material and solvent, facilitating release of protein. As shown in Figure 2D, the time course of chitin extraction using LysMEA at 120 °C and a solid-to-liquid ratio of 1:20 influenced the process to reach equilibrium at 12 h. An extension of time did not reduce the DP rate, indicating its effective protection of chitin and preventing excessive depolymerization. For the convenience of expression, the chitin product prepared under the optimized condition for 12 h was designated as LM-chitin.

**Figure 3:**
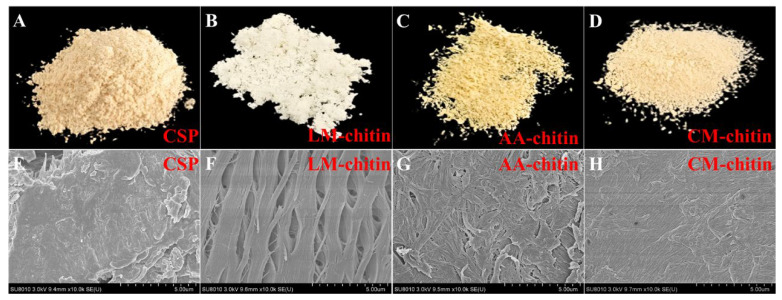
Appearance and micromorphology of chitin samples. Photographs (**A**–**D**) and SEM images (**E**–**H**) of CSP and chitin extracted by LysMEA, acid-alkali method and ChClMA (denoted as LM-chitin, AA-chitin and CM-chitin, respectively).

The reusability of LysMEA was investigated over 5 cycles of chitin extraction from DM-CSP. A limited number of reuse cycles did not affect the DP efficiency of DES, with the DP rate still reaching 96.4% after five cycles (Figure S3). As the number of reuse cycles increased, the color of the DES became progressively darker, eventually exhibiting a dark red hue after five cycles. Unlike the pale yellow color of CSP, LM-chitin is white, indicating the release of pigments from the raw material into the solvent (Figure 3A,B). The phenomena suggest that the LysMEA DES system is effective not only in deproteinizing but also in decolorizing crayfish shell waste, making it suitable for the preparation of high-purity chitin. This result is similar to the findings of Zhang et al. [33].

Unlike traditional methods that require separate, aggressive treatments, the LysMEA system simultaneously removes proteins and modifies the chitin architecture in a single, recyclable solvent system. Although the overall process is not entirely ‘solvent-based’ due to the initial acid step, the replacement of the conventional high-temperature, high-concentration alkaline DP step with this recyclable DES significantly reduces the environmental burden. Furthermore, as demonstrated in the following sections, this approach uniquely produces chitin with superior enzymatic reactivity, which is the primary value of this strategy.

### 3.3. Structural Characterization and Comparison

Currently, the acid-alkali and acidic DES extraction methods are widely used for chitin preparation from bioresources. To compare the chitin extracted from crayfish shell waste using alkaline LysMEA DES with that obtained via the acid-alkali and acidic ChClMA DES (components, see Table 1) extraction (designated as AA-chitin and CM-chitin, respectively), this study prepared and systematically characterized all three products. The chitin obtained through one-step ChClMA treatment exhibited a DP rate of only 87.8% (Figure S2), thereby necessitating an additional NaOH treatment to enhance its purity. As shown in Table 2, LM-chitin displayed a purity comparable to that of commercial chitin (97.1% vs. 97.4%), and was purer than AA-chitin, CM-chitin and chitins extracted by other DES systems, such as ChCl-malonic acid (93.0%), ChCl-formic acid (93.4%) and proline-urea-DBU (92.5%) [33,52,53]. As mentioned, LM-chitin was white, while AA-chitin and CM-chitin were similar in color to CSP (Figure 2A–D). This indicates that the two-step extraction method developed here can be used to prepare chitin with fewer impurities, traditionally produced via three-step processes of DM, DP and decolorization [54]. Moreover, a comparable chitin yield (>70%) was observed between LysMEA extraction and chemical preparation, both of which were higher than that obtained by ChClMA treatment (Table 2). These results highlight the potential of the LysMEA system for the production of high-purity chitin from crayfish processing waste.

**Figure 4:**
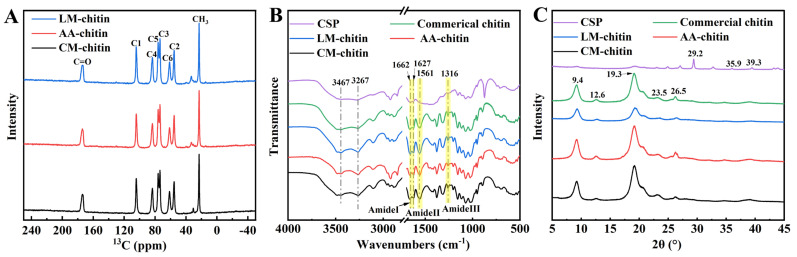
The ^13^C CP/MAS NMR (**A**), FTIR (**B**) and XRD (**C**) spectroscopy of various chitin samples.

The chemical structure of the extracted chitins was confirmed by ^13^C CP/MAS NMR and FTIR analysis. As illustrated in Figure 4A, the samples exhibited ^13^C signals at 23.1, 55.4, 61.4, 73.7, 76.1, 83.5, 104.4, and 174.1 ppm, which were attributed to the methyl, C2, C6, C3, C5, C4, C1, and carbonyl carbons, respectively. In their NMR spectra, the distinct splitting of the C3 and C5 peaks indicates that the resulting products were all α-chitins [55]. Furthermore, the FTIR spectra of these three chitins, which closely resemble that of commercial chitin (Figure 4B), provide additional corroboration for this conclusion. An amide I band of α-chitin, characterized by two peaks at 1662 and 1627 cm^−1^, was observed in their spectra [53]. The broad peaks at 3467 and 3267 cm^−1^ were attributed to O−H and N−H stretching vibration, respectively. Two peaks at 1561 and 1316 cm^−1^, representing amide II and III bands, were also found for each chitin sample. The amide I band of untreated CSP did not exhibit splitting, due to the overlap between the protein and chitin amide C=O stretching signals. In contrast, the amide I bands of the four chitin samples showed clear separation, indicating the relatively complete removal of protein [13]. In addition, the degree of deacetylation (DD) of extracted chitins was determined from their FTIR spectra (Table 1). The DD values of LM-chitin and AA-chitin were similar to commercial chitin but significantly lower than CM-chitin, suggesting that the alkaline DES had a limited effect on chitin deacetylation. The DD values for LM-chitin, AA-chitin, and CM-chitin (17.4%, 13.0%, and 23.1%) were generally consistent with those calculated using the ^13^C CP/MAS NMR method (16.1%, 11.2%, and 22.9%). This result differs from the ternary alkaline DESs developed by Zhang et al. [33].

XRD patterns of LM-chitin, AA-chitin, CM-chitin and CSP were measured to determine their crystallinity. The crystal reflections at 2θ = 9.4°, 12.6°, 19.3°, 23.5°and 26.5° of chitins obtained by LysMEA, ChClMA and chemical treatment were in good agreement with those of commercial chitin (Figure 4B). All these samples had characterized diffraction peaks of α-chitin [56]. The diffraction peaks of CSP displayed crystal reflections of CaCO_3_ at 2θ = 29.2°, 35.9°, and 39.3°, whereas the patterns of extracted chitins did not exhibit these peaks, thereby confirming the high purity of the chitins extracted using both DESs and the chemical method. Furthermore, the crystalline index (CrI) of LM-chitin was lower than that of AA-chitin and commercial chitin (68.3%, 86.5% and 86.1%, respectively), implying the capacity of the alkaline DES system to disrupt the crystalline structure of chitin. Acidic DESs have been demonstrated to possess strong hydrolytic activity towards the amorphous regions of chitin, thereby enhancing its crystallinity. Consequently, the crystallinity of CM-chitin (90.2%) was significantly higher than that of the other samples.

The molecular weight (M_w_) of extracted and commercial chitins is shown in Table 2. LM-chitin exhibited a M_w_ of 203.9 kDa, significantly higher than that of AA-chitin (122.8 kDa) and CM-chitin (77.1 kDa). The result suggests that the LysMEA system effectively prevents chitin degradation during extraction, maintaining a higher molecular weight of the product. Alkaline DES systems have also demonstrated a comparable ability to maintain molecular weight during cellulose extraction [57].

The morphologies of CSP and the extracted chitins were examined by SEM. As shown in Figure 3E, the CSP particles exhibited an irregular block-shaped and relatively flat appearance. In crayfish shells, CaCO_3_ serves as a matrix within the chitin–protein fibers, with the three substances closely bonded to form a compact structure. After sequential treatment with HCl and LysMEA, the chitin product from CSP displayed a loose mesh-like structure (Figure 3G). This morphological observation is consistent with the disruption of the mineral–protein matrix. Compared to LM-chitin, the chitin fibers in AA-chitin were more densely packed, although some amorphous regions were exposed on the surface (Figure 3G). In contrast, CM-chitin treated with acidic DES displayed a smooth and uniform surface (Figure 3H), which may suggest a loss of amorphous material. The microstructure of CM-chitin resembled that of chitin extracted by other acidic DESs [40,58]. Collectively, these SEM observations, when combined with the XRD and BET (discussed blow) measurements, provide a comprehensive picture of the structural differences between the samples.

The BET tests were executed to confirm the change in the surface and pore structure of the extracted chitins. Their nitrogen sorption isotherms, pore size distributions and adsorption cumulative pore volumes are illustrated in Figure S4. The average pore sizes of LM-chitin, AA-chitin and CM-chitin are 12.7 nm, 22.1 nm and 11.6 nm, respectively (Table 2), indicating a mesoporous structure. Their pore volumes are positively correlated with their average pore sizes. As expected, the BET surface aera of LM-chitin (15.1 m^2^/g) is superior to AA-chitin and CM-chitin (6.7 m^2^/g and 8.0 m^2^/g). In the SEM images, the surface of AA-chitin is significantly rougher than that of CM-chitin, yet its surface area is slightly lower, possibly due to the larger but fewer pores on AA-chitin. In contrast, LM-chitin presents a larger surface area and smaller pore sizes, indicating a more porous structure that facilitates better contact with enzymes or chemical reagents. The surface area of LM-chitin is larger than the chitins treated with instant catapult steam explosion, concentrated HCL and ultrasonication, further demonstrating the ability of the LysMEA system to enhance the accessibility of chitin [20,22,59].

### 3.4. Enzymatic Reactivity

**Figure 5:**
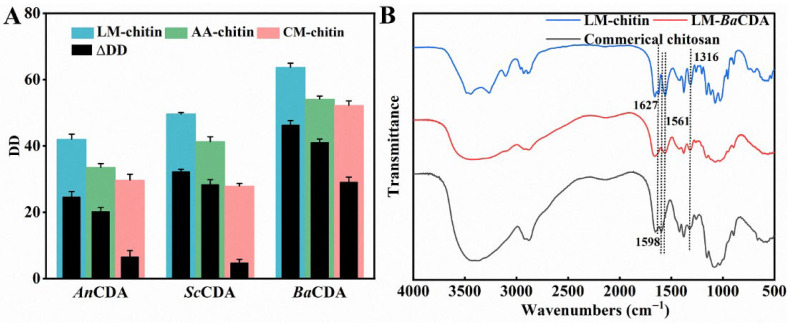
Catalytic efficiency of different CDAs on various chitin substrates. The increase in degree of deacetylation (DD) of chitin substrates after 12 h of reaction catalyzed by different CDAs (**A**). The FTIR spectra of commercial chitosan, LM-chitin, and deacetylation product of LM-chitin generated by BaCAD catalysis (named CTS-LM-Ba) (**B**).

To evaluate the functional impact of structural engineering, the accessibility of the chitins to enzymes was tested using three recombinant chitin deacetylases (CDAs) from *Aspergillus nidulans*, *Bacillus aryabhattai* and *Saccharomyces cerevisiae* (termed as AnCDA, BaCDA and ScCDA, respectively), all of which are active against colloidal chitin. The deacetylation effects of these enzymes on the chitin samples were subsequently evaluated. As shown in Figure 5A, all three enzymes exhibited the highest deacetylation efficiencies on LM-chitin, with AnCDA, BaCDA, and ScCDA increasing their DD by 24.9%, 46.6%, and 32.6%, respectively. The deacetylation product of LM-chitin catalyzed by BaCDA (denoted as CTS-LM-Ba) achieved a DD of 63.7%, with a solubility of 68.9 ± 0.8% in a 1% acetic acid solution, demonstrating typical chitosan characteristics [60]. The comparison of the FTIR spectra of LM-chitin, CTS-LM-Ba and commercial chitosan also supports the observation. As illustrated in Figure 5, the characteristic amide I band of LM-chitin at 1627 cm^−1^ disappeared in both chitosan samples. However, distinct differences were observed in the amide II region. Unlike commercial chitosan, which exhibited a characteristic peak at 1598 cm^−1^, CTS-LM-Ba retained the N-H bending vibration peak at 1561 cm^−1^ found in LM-chitin. Furthermore, the amide III band at 1316 cm^−1^ was significantly weaker in CTS-LM-Ba compared to LM-chitin, showing an intensity similar to that of commercial chitosan. Collectively, these spectral changes indicate that BaCDA effectively catalyzes the deacetylation of the chitin backbone [61]. However, the retention of the 1561 cm^−1^ band suggests that the N-deacetylation is not exhaustive, resulting in a partially deacetylated product.

The superior deacetylation performance of CDAs on LM-chitin is attributed to a synergistic combination of structural modifications induced by the LysMEA extraction process. First, the significantly reduced CrI of LM-chitin (68.3%, compared to 86.5% for AA-chitin and 90.2% for CM-chitin) plays a pivotal role. The dense crystalline regions in native chitin act as a physical barrier, restricting enzyme diffusion and binding. The disruption of the inter- and intra-chain hydrogen bonding network by LysMEA (as confirmed by MD simulations in Section 3.5) results in a more amorphous structure, which is inherently more susceptible to enzymatic attack. Second, the increased specific surface area of LM-chitin (15.1 m^2^/g, compared to 6.7 m^2^/g for AA-chitin and 8.0 m^2^/g for CM-chitin) provides a larger contact area for the enzyme–substrate interaction. This enlarged surface area allows for a higher density of enzyme adsorption, facilitating the initial binding step of the catalytic process. Finally, the altered pore structure observed in the SEM images and the shift in pore size distribution contribute to this enhancement. The loose mesh-like structure and mesoporous architecture of LM-chitin likely facilitate the mass transfer of the enzyme molecules into the substrate matrix, reducing steric hindrance. In contrast, the highly compact and smooth surface of CM-chitin and the larger but fewer pores of AA-chitin limit the accessibility of the catalytic pockets within the chitin fibers. Therefore, it is the concerted effect of these structural parameters, lower crystallinity, higher surface area, and favorable pore morphology, that renders LM-chitin highly reactive towards enzymatic deacetylation.

While the enzymatic deacetylation efficiency of LM-chitin is significantly enhanced, it is important to acknowledge that this study primarily serves as a proof-of-concept. The current work focuses on demonstrating the feasibility of combining DES with enzymatic catalysis. For true industrial translation, several practical aspects require further optimization in future studies. These include the cost-effectiveness of the LysMEA solvent recovery, the evolution of the enzyme, and its high-efficiency production and recyclability. Addressing these challenges will be crucial for determining the economic viability of this integrated process.

### 3.5. Molecular Origins of Chitin Disordering Caused by Solvent Treatment

**Figure 6:**
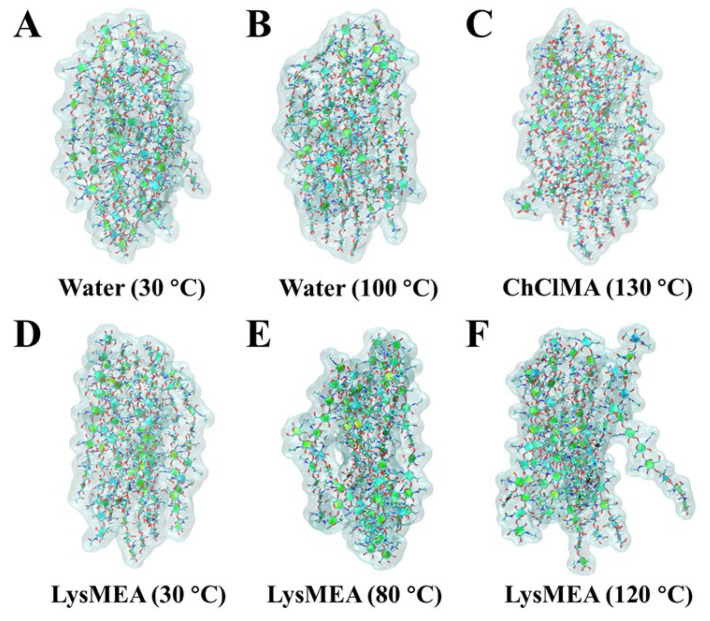
Snapshots of chitin fiber bundles immersed in different solvents at varying temperatures after 200 ns of simulation. Water systems at 30 °C (**A**) and 100 °C (**B**), ChCLMA system at 130 °C (**C**), and LysMEA systems at 30 °C (**D**), 80 °C (**E**) and 120 °C (**F**). Chitin fibers and each chitin chain are shown as surface and stick, respectively.

**Figure 7:**
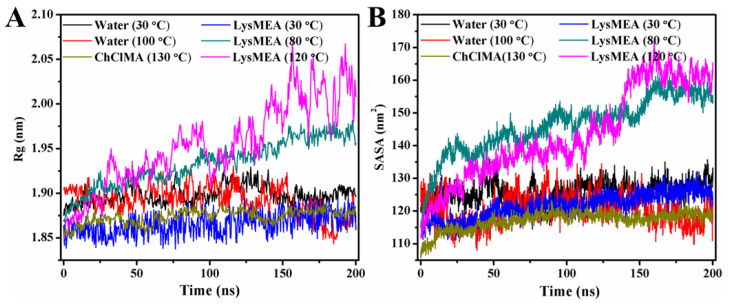
Analysis of MD simulation results. Time course of radius of gyration (Rg) (**A**) and solvent-accessible surface area (SASA) (**B**) of chitin fiber bundles immersed in different environments.

To elucidate the molecular mechanism behind the chitin disintegration observed experimentally, models of chitin fiber bundles immersed in water, LysMEA, and ChClMA were constructed, followed by subsequent molecular dynamics (MD) simulations of each model at varying temperatures for 200 ns. As shown in Figure 6A–D, simulating a chitin fiber bundle in water at 303 K (30 °C) and 373 K (100 °C), in ChCLMA at 403 K (130 °C), as well as in LysMEA at 30 °C, did not lead to significant changes in its compact structure within 200 ns. Figure 7A,B illustrate the trends in the radius of gyration (Rg) and solvent-accessible surface area (SASA) of a chitin fiber bundle in these environments, respectively. During the simulations, Rg and SASA of chitin were maintained in both ambient and boiling water, and even in ChCLMA at 130 °C, reflecting its recalcitrant nature across a wide range of temperatures. In a previous MD study, chitin slab also exhibited the same stability in the ChCL-lactic acid DES system at 450 K [62]. However, the regular arrangement of the chitin chains was disrupted when positioned in LysMEA at 353 K (80 °C) and 393 K (120 °C) within 200 ns of simulation (Figure 6E,F). The trends in Rg and SASA of the chitin fiber bundle remained stable in LysMEA at 30 °C. On the contrary, these parameters progressively increased with the extension of the simulation time at 80 °C and 120 °C and displayed a temperature-dependent behavior (Figure 7A,B). The theoretical prediction indicates that incubating chitin in LysMEA at 120 °C can enhance its disorder and surface area, which align with the results obtained from XRD and BET analysis.

**Figure 8:**
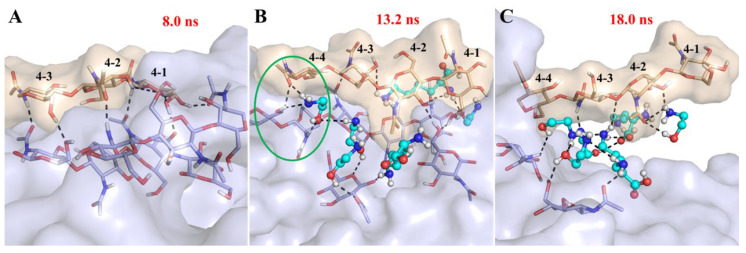
Dissociation process of the outer chain of a chitin fiber bundle in LysMEA at 120 °C. Snapshots of the outer chain No. 4 (wheat) tightly bound to the core (lightblue) (**A**), solvent penetration (**B**), and partial dissociation of the outer chain (**C**). Chitin fiber and solvent molecules are represented as surface and ball-and-stick, respectively. Hydrogen bonds, along with the chitin units involved in their formation, are depicted as black dash and stick, respectively. The carbon atoms have the same color as the surface, while the oxygen, nitrogen and hydrogen atoms are shown in red, blue and white respectively. The green circle in (**B**) represents the solvent molecules weakening the interchain interactions of chitin through hydrogen bonding.

Figure 8 exhibits the detailed process of chitin chain peeling in LysMEA at 120 °C. At the initial stage of the simulation, one side of the outer chain was in close contact with the core part of the chitin fiber bundle by interchain H-bonds (Figure 8A). As Lys and MEA molecules progressively penetrated between the chitin chains, the separation of the outer chain began. The temperature-dependent weakening of interchain interaction within the chitin fiber provided a chance for the competitive formation of new H-bonds between solvent molecules and the polar groups involved in interchain hydrogen bonding (Figure 8B). This process leads to the partial dissociation of outer chains from the fiber bundle (Figure 8C), resulting in the observed reduction in crystallinity and an increase in accessibility. A similar but slower process was observed in the same system at 80 °C.

Although the total number of H-bonds in the LysMEA system was similar across temperatures (Table 3), the stronger intermolecular H-bond interactions and longer bond lifetimes observed at 30 °C indicate an inert molecular state that hindered solvent penetration. In contrast to the LysMEA system, the behavior of other solvents differed significantly. In an aqueous system, despite the sufficient number of H-bonds formed between water and chitin at 100 °C, these interactions were too unstable to facilitate penetration. Conversely, in the ChClMA system, although stable H-bonds were formed (lifetimes of 0.94 ns for choline-chitin and 1.82 ns for malic acid-chitin), its viscosity was orders of magnitude higher than that of LysMEA (Figure 1B). This high viscosity, resulting from extensive H-bonding between solvent molecules, likely restricted solvent–chitin interactions. These observations suggest a dual requirement for effective penetration. Chitin requires higher temperatures to weaken interchain interactions, while the solvent must form relatively stable H-bonds with chitin to compete for binding sites. Previous studies on ILs support this, indicating that ions with stronger charges are more effective at disrupting chitin structure [62,63]. Therefore, these findings provide a rational design principle for developing novel solvent systems that enable structural modification of chitin at lower temperatures.

## 4. Conclusions

This study presents a novel and integrated strategy for the extraction and structural engineering of chitin from red swamp crayfish shell waste using an alkaline DES system composed of Lys and MEA. The optimized process yielded high-purity chitin (97.1%) with a well-preserved molecular weight (203.9 kDa). Crucially, the system effectively disrupted the crystalline architecture of chitin, resulting in a porous, low-crystallinity structure that was highly accessible to enzymes. This structural engineering facilitated highly efficient enzymatic deacetylation, yielding a DD of 63.7%, which markedly outperformed chitin derived from conventional acid-alkali or acidic DES methodologies. MD simulations confirmed that the mechanism involves solvent penetration and the disruption of interchain hydrogen bonds at elevated temperatures. This strategy demonstrates the feasibility of integrating extraction with structural modification to facilitate biocatalytic valorization of crustacean shell waste.

## Figures and Tables

**Table 1:** Initial DES components used for screening.

HBA	HBD	Molar Ratio (HBA: HBD)	Designation
ChCl	MEA	1:4	ChClMEA
ChCl	TEA	1:4	ChClTEA
Asp	MEA	1:4	AspMEA
Asn	MEA	1:4	AsnMEA
Gln	MEA	1:4	GlnMEA
Pro	MEA	1:4	ProMEA
Lys	MEA	1:4	LysMEA
Lys	TEA	1:4	LysTEA
K_2_CO_3_	glycerol	1:4	KG
ChCl	MA	1:2	ChClMA

**Table 2:** Purity, yield, molecular weight (M_w_), degree of deacetylation (DD), crystalline index (CrI), BET surface areas, pore volume and average pore size of extracted chitin from red swamp crayfish waste.

Sample Name	Purity (%)	Yield (%)	DD (%)	CrI (%)	M_w_ (kDa)	BET Surface Areas (m^2^/g)	Pore Volume (cm^3^/g)	Average Pore Size (nm)
Commercial chitin	97.4 ± 0.4	-	12.4 ± 0.2	86.1 ± 0.6	501.3 ± 2.5	-	-	-
LM-chitin	97.1 ± 0.1	70.8 ± 1.4	17.4 ± 0.5	68.3 ± 1.3	203.9 ± 0.5	15.1	0.039	12.7
AA-chitin	95.3 ± 0.3	75.1 ± 1.1	13.0 ± 0.4	86.5 ± 2.2	122.8 ± 0.2	6.7	0.054	22.1
CM-chitin	95.1 ± 0.3	64.0 ± 1.2	23.1 ± 0.6	90.2 ± 1.3	77.1 ± 0.7	8.0	0.032	11.6

**Table 3:** Statistical analysis of H-bonds formed between chitin and solvent molecules.

Solvent	Temperature (°C)	H-Bond Type ^a^	H-Bond Number	H-Bond Life (ns)
Water	30	H_2_O-Chitin	512.4 ± 14.8	0.12
	100	H_2_O-Chitin	441.7 ± 17.6	0.02
LysMEA	30	Lys-Chitin	101.7 ± 11.5	4.25
		MEA-Chitin	243.0 ± 13.6	1.27
	80	Lys-Chitin	113.9 ± 11.6	1.22
		MEA-Chitin	256.2 ± 20.4	0.46
	120	Lys-Chitin	101.4 ± 13.4	0.30
		MEA-Chitin	213.8 ± 20.7	0.15
ChClMA	130	CHO-Chitin	12.8 ± 3.2	0.94
		MA-Chitin	63.2 ± 8.0	1.82

^a^ H-bonds formed between assigned molecules.

## Data Availability

The original contributions presented in this study are included in the article/Supplementary Materials. Further inquiries can be directed to the corresponding authors.

## Supplementary Materials

The following supporting information can be downloaded at: https://www.mdpi.com/article/10.3390/foods15071159/s1, Figure S1. Formation of the initial model for chitin fiber bundle. Original model constructed by Packmol (A). Model undergoing 50 ns pre-balance (B). Figure S2. Demineralization (DM) and deproteinization (DP) rate of chitin extracted from red swamp crayfish shell powder (CSP) using various DESs. Alkaline DES extraction was performed at 1:15 of solid-to-liquid ratio and 100 °C for 12 h, and ChClMA DES extraction was conducted at 1:20 of solid-to-liquid ratio and 130 °C for 3 h. Figure S3. Recycling of LysMEA (Lys: MEA = 1:8) in chitin extraction using demineralized crayfish shell powder (DM-CSP) as raw material. Treatment conditions: extraction temperature of 120 °C, solid-to-liquid ratio of 1:20 (*w*/*w*) and treatment time of 12 h. Figure S4. Nitrogen sorption isotherms, pore size distributions and adsorption cumulative pore volumes of LM-chitin (A–C), AA-chitin (D–F) and CM-chitin (G–I). References [64,65,66] are cited in Supplementary Materials.

## Abbreviations

DES: deep eutectic solvent; DM: demineralization; DP: deproteinization; IL: ionic liquids; CDA: chitin deacetylase; DBU: 1,8-diazabicyclo [5.4.0] undec-7-ene; MEA: monoethanolamin; ChCl: choline chloride; TEA: triethanolamine; DMAc: N,N-dimethylacetamide; MA: malic acid; IPTG: isopropyl β-D-1-thiogalactopyranoside; CSP: crayfish shell powder; DM-CSP: demineralized crayfish shell powder; HBA: hydrogen bond acceptor; HBD: hydrogen bond donor; FTIR: Fourier-transform infrared spectroscopy; M_w_: average molecular weight; DD: deacetylation degree; CrI: crystalline index; SEM: scanning electron microscope; BET: Brunauer–Emmett–Teller analysis; LB: Luria–Bertani; Molecular dynamics: MD.
