# Supplementary material for: Integrated Extraction and Structural Engineering of Chitin from Crayfish Shell Waste Using Alkaline Deep Eutectic Solvents Toward Facile Enzymatic Deacetylation
Source: Foods. 2026 Mar 30;15(7):1159. doi: 10.3390/foods15071159 (PMC13073664; doi:10.3390/foods15071159)
Supplement: Supplementary file 1 [file foods-15-01159-s001.zip › foods-4217885-supplementary.pdf]

Supporting Information

Integrated extraction and structural engineering of chitin from crayfish shell waste using alkaline deep eutectic solvents toward facile enzymatic deacetylation

Zexin Zhao, Shengyu Yang, Kaige Chen, Haojie Zhang, Jun Cai*

Key Laboratory of Fermentation Engineering (Ministry of Education), National "111" Center for Cellular Regulation and Molecular Pharmaceutics, Hubei Key Laboratory of Industrial Microbiology, Cooperative Innovation Centre of Industrial Fermentation (Ministry of Education & Hubei Province), Hubei University of Technology, Wuhan 430068, PR China

1. **Computational methods**
2. **Other supplementary Tables and Figures**

* To whom correspondence should be addressed:

Prof. Jun Cai E-mail: hgdcaijun@hbut.edu.cn

1. Computational methods

1.1 Generation of chitin fiber bundle model

The initial structure of the chitin decamer was generated using the *tleap* module of AmberTools and subsequently minimized under vacuum with the GLYCAM06j-1 force field [1]. An incompact chitin fiber, consisting of twelve chitin decamers, was constructed using Packmol [2] and centrally placed within a periodic water box, ensuring a buffer distance of 15 Å (the initial system sees Fig. S1A). The TIP3P water model was employed to represent the solvent molecules. The system underwent multiple minimization steps to achieve structural relaxation, followed by a 100 ps molecular dynamics (MD) simulation under the NVT ensemble to heat it to a target temperature of 303 K. Subsequently, an additional 100 ps MD simulation was conducted under the NPT ensemble, with the target temperature and pressure set to 303 K and 1.0 atm, to equilibrate the density of the system to approximately 1.0 g/cm^3. The densely aggregated and stable chitin chains (self-assembly), obtained after a 50 ns NPT MD simulation with a time step of 2.0 fs, served as the initial model for chitin fiber bundle (Fig. S1B). Throughout the simulation, the LINCS algorithm was employed to constrain hydrogen bonds. The V-rescale method and the Parrinello–Rahman barostat were used to regulate the temperature and pressure of the simulation system, respectively. Additionally, a 14 Å cutoff was applied for both van der Waals and electrostatic interactions. All MD simulations in the study were conducted using GPU-accelerated engine provided by GROMACS 2019 [3]. Except for the target temperature and number of MD-steps, the other settings are the same as mentioned above.

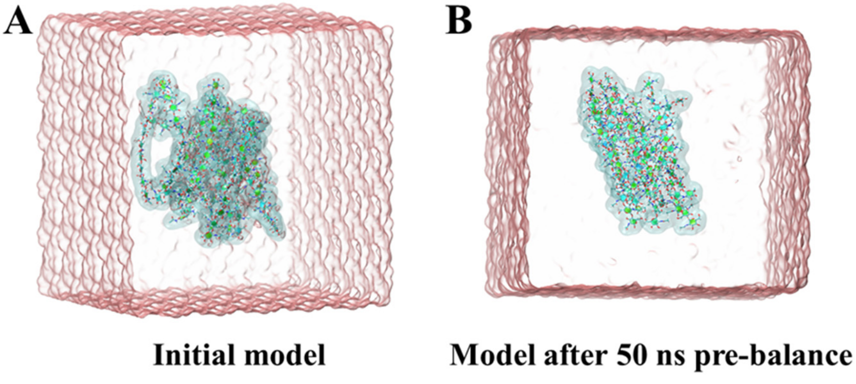

Fig. S1. Formation of the initial model for chitin fiber bundle. Original model constructed by Packmol **(A)**. Model undergoing 50 ns pre-balance **(B)**.

1.2 Construction of DES solvent treatment systems for chitin fiber bundle

The optimized structures of L-lysin (Lys), monoethanolamine (MEA), L-malic

acid, D-malic acid and choline were acquired from the ATB website (https://atb.uq.edu.au/index.py). The force field parameters for these molecules were generated using the AMBER GAFF force field [4]. The restrained electrostatic potential (RESP) charges, calculated at the B3LYP/6-311G (d, p) level using Gaussian 16, were assigned as the partial atomic charges for each molecule. Chloride ions were described by the Amber99SB-ildn force field [5]. For the LysMEA (molar ratio of Lys to MEA = 1:8) system, the previously prepared chitin fiber bundle was first placed at the center of a periodic box with dimensions of 10 × 10 × 10 Å. The empty space was then randomly filled with 900 Lys and 7200 MEA molecules. Additionally, the Lys and MEA molecules in LysMEA system were replaced with 1800 L-malicacid, 1800 D-malicacid, 1800 choline molecules, and 1800 chloride ions to gain the ChClMA (molar ratio of ChCl to malicacid = 1:2) system. Both systems were constructed using Packmol software.

1.3 MD simulations of different systems

The water treatment system directly utilized the final structure obtained from Section 1 as the initial model. An additional 200 ns NPT MD simulation was performed at 303 K to predict the structural evolution of the chitin fiber bundle under ambient temperature. Meanwhile, the initial model underwent sequential 100 ps NVT and NPT MD simulations to heat the system to 373 K and equilibrate its density. Subsequently, a 200 ns NPT simulation was conducted to produce the trajectory of the chitin fiber bundle in boiling water. For the LysMEA and ChClMA systems, 100 ps NVT and 2 ns NPT MD simulations were carried out to gradually raise the temperature to a certain value and equilibrate the system density. As with the water system, a 200 ns NPT MD simulation was then conducted for each DES system to investigate the effects of temperature on the ability of DES to disaggregate the chitin fiber bundle in *silico*.

References

1. Kirschner, K.N.; Yongye, A.B.; Tschampel, S.M.; González-Outeiriño, J.; Daniels, C.R.; Foley, B.L.; Woods, R.J. Glycam06: A generalizable biomolecular force field. Carbohydrates. *Journal of Computational Chemistry.* **2008**, 29, 622-655.
2. Martínez, L.; Andrade, R.; Birgin, E.G.; Martínez, J.M. Packmol: A package for building initial configurations for molecular dynamics simulations. *Journal of Computational Chemistry.* **2009**, 30, 2157-2164.
3. Abraham, M.J.; Murtola, T.; Schulz, R.; Páll, S.; Smith, J.C.; Hess, B.; Lindahl, E.

Gromacs: High performance molecular simulations through multi-level parallelism from laptops to supercomputers. *SoftwareX.* **2015**, 1-2, 19-25.

4. Wang, J.; Wolf, R.M.; Caldwell, J.W.; Kollman, P.A.; Case, D.A. Development and testing of a general amber force field. *Journal of Computational Chemistry.* **2004**, 25, 1157-1174.
5. Lindorff-Larsen, K.; Piana, S.; Palmo, K.; Maragakis, P.; Klepeis, J.L.; Dror, R.O.; Shaw, D.E. Improved side-chain torsion potentials for the amber ff99sb protein force field. *Proteins: Structure, Function, and Bioinformatics.* **2010**, 78, 1950-1958.

2. Other supplementary Tables and Figures

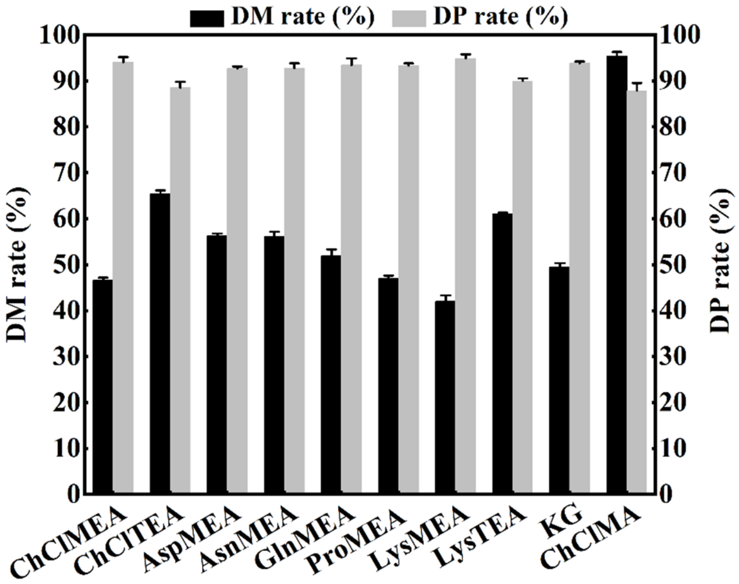

Fig. S2. Demineralization (DM) and deproteinization (DP) rate of chitin extracted from red swamp crayfish shell powder (CSP) using various DESs. Alkaline DES extraction was performed at 1:15 of solid-to-liquid ratio and 100 °C for 12 h, and ChClMA DES extraction was conducted at 1:20 of solid-to-liquid ratio and 130 °C for 3 h.

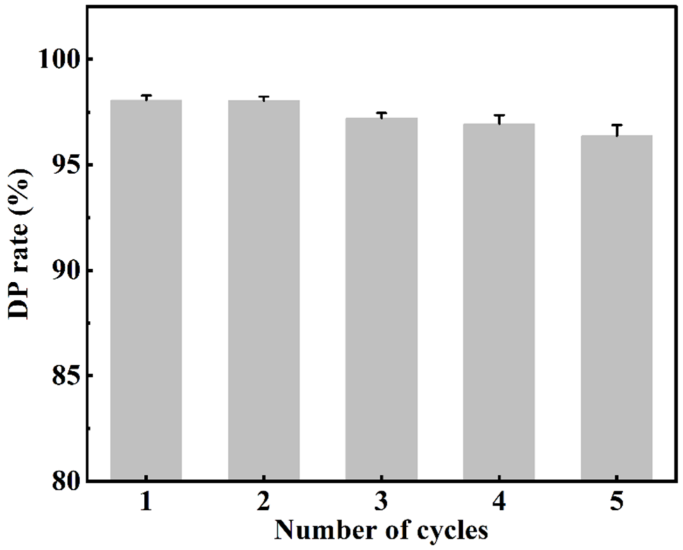

Fig. S3. Recycling of LysMEA (Lys: MEA = 1:8) in chitin extraction using demineralized crayfish shell powder (DM-CSP) as raw material. Treatment conditions: extraction temperature of 120 °C, solid-to-liquid ratio of 1:20 (w/w) and treatment time of 12 h.

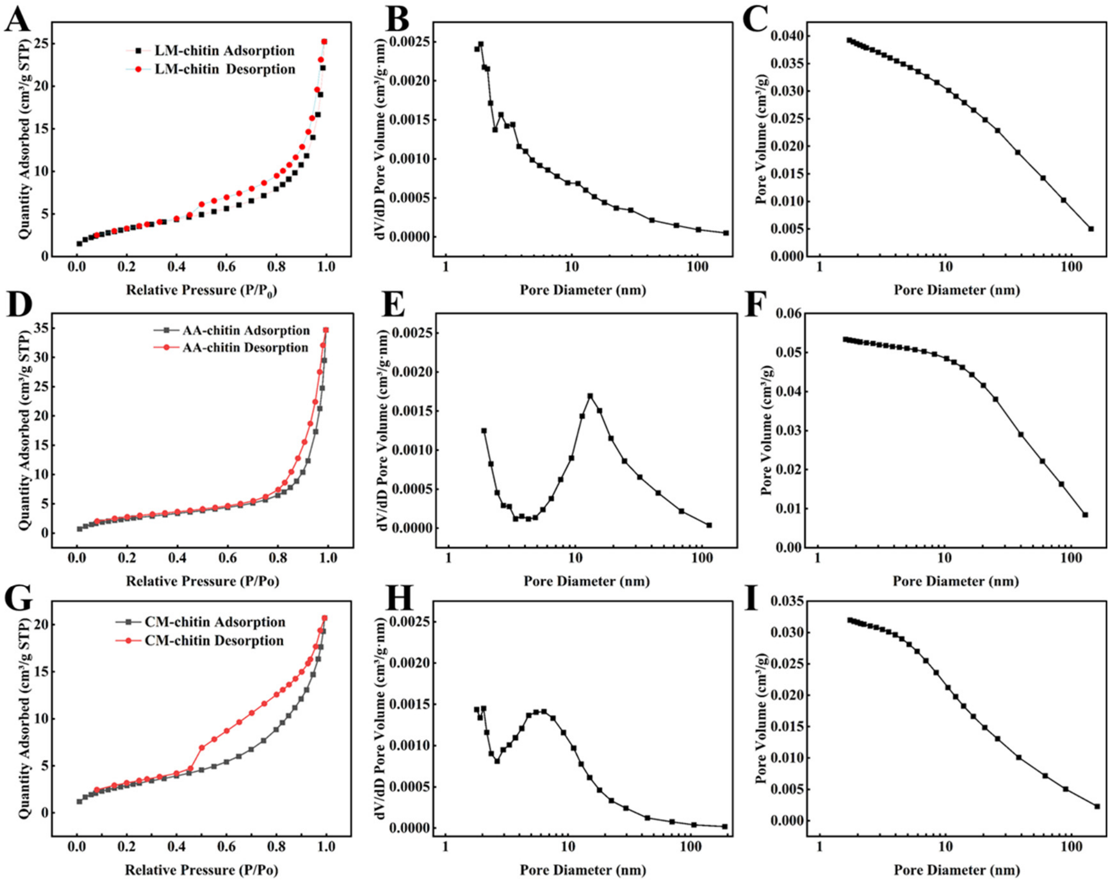

Fig. S4. Nitrogen sorption isotherms, pore size distributions and adsorption cumulative pore volumes of LM-chitin **(A-C)**, AA-chitin **(D-F)** and CM-chitin **(G-I)**.
